# Hospitals and the Law in 1910

**Published:** 1911-01-21

**Authors:** 


### HOSPITALS AND THE LAW IN 1910.

### I.—HOSPITALS AND LICENCE DUTIES.

Signs are not wanting that those who are entrusted with the invidious task of collecting revenue are prepared to pursue their full legal remedies against hospitals and other charitable institutions. A case which very nearly affects the interests of every hospital which employs porters, gardeners, etc., was recently considered and decided at Otley.

### The Leeds Hospital Fund Case.

The facts lie in very small compass. It appears that a society known as the Leeds Workpeople's Hospital Fund supports (amongst other institutions) a convalescent home for Leeds workmen at Ilkley. In the grounds of the home a gardener named William Henderson is employed. Now the Revenue Act of 1896, s. 18, imposes a duty of 15s. on a licence to employ a male servant, and the duty of collecting this tax is imposed upon the local authority. Seeing a male servant so employed in the gardens of the home, the West Riding Local Taxation Licences Committee were minded to collect a tax in respect of him. The "Fund" refused to pay; whereupon an information was laid against Walter Wormald, the Secretary of the Convalescent Home, the charge being that on a certain date "he failed to take out a licence in respect of the employment of a male servant."

### The Liability of a Hospital Secretary.

Stress was laid on the fact that in a former year a licence had been taken out in respect of this man. The Fund was a limited company, and the gardener was employed by the Fund and not by the Secretary. The Secretary, in the course of his evidence, said he had no powers to employ or dismiss any of the servants working for the Fund. One of the senior magistrates for Leeds, who was the founder of the Fund and had been its Chairman from the start, said it was within his knowledge that medical charities in Leeds did not pay licences for male servants, and having that knowledge he had a *boná-fide* belief that the Fund was not liable. The judgment of the Bench (pronounced by the Chairman) was as follows: "The case has been interesting, and I can quite understand that it is of general importance. We are quite clear in our minds that Henderson is a gardener, and as such is subject to licence duties. On the other hand, we are clearly of opinion that the Secretary is not liable in his own proper person, and that the Committee of the Fund, which is liable, is not before the Court. In these circumstances, the case will be dismissed."

The case was dismissed—subject to the statement of a special case and possible further argument before the judges of the King's Bench Division. The reasons why it was dismissed cannot be said to be altogether satisfactory to hospital authorities. An information against the Committee of the Fund is bound to be successful if the magistrate's view of the law is correct.

It becomes important, therefore, to consider whether employers are really liable in respect of persons engaged to do the work upon which this gardener was employed.

### Male Servants in Hospitals.

The liability to pay licence duty for male servants arises under the Revenue Act of 1869, which, by Section 18, imposes a duty of 15*s.* on a licence to employ a male servant. The term "male servant," as defined by the following section, is a comprehensive one, including all such servants as butler, footman, cook, porter, groom, helper in the stables, gardener and under-gardener, huntsman, or persons employed "in any capacity involving the duties of any of the above descriptions of servants, by whatever style the persons acting in such capacity may be called." So sweeping, indeed, was the description that it was found necessary to provide a modification in the Customs and Inland Revenue Act, 1876, which enacts by Section 5 that the term "male servant" shall not include "a servant who, being *boná-fide* employed in any capacity other than the capacities specified or referred to in the previous Act, is occasionally or partially employed in any of such capacities, nor a person who has been *boná-fide* engaged to serve his employer for a portion only of each day, and does not reside in his employers' premises?"

### Who is an "Employer"?

The Acts, apparently, do not define employer; and the burden may, so far as one can see, be imposed upon charitable organisations as well as other employers. It appears, however, that the judges have desired to restrict the operation of the Act as far as possible to private employers. In the case of Whiteley *v.* Burns, which was referred to when the case under review was being argued, Messrs. Whiteley, the Universal Providers, appealed against an order to pay licence duty on a number of men employed in their establishment. They have a very large number of assistants, who have breakfast, dinner, tea, and supper on the premises. For this purpose Messrs. Whiteley employed thirty-five men, one of whom was a superintendent, four cooks, and the rest waiters. In that case it was held that these thirty-five men did not come within the definition of male servants under the Revenue Acts, which refer, it was said, primarily to private establishments, and not to trade servants. This is some consolation for trading firms, and the second enactment quoted above seems to show that the ordinary "handy-man," who does odd jobs, from carrying coals to cutting the hedge and cleaning the knives, is not within the Act. Whether the High Court of Justice will endeavour to make an exception in favour of hospitals and other charitable institutions remains to be seen. Unless the law be so interpreted it is manifest that the hospital and the charitable institution will be in a position less favourable than that of the shopkeeper and the small tradesman. In Burns' case

(*supra*) the Lord Chief Justice points out that the intention of the legislature was to tax luxuries by a kind of "sumptuary law."

We have yet to learn that the employment of necessary servants by a medical charity is the indulgence of a luxury.

Hospitals and Armorial Bearings.

The other case to which we desire to refer is one which came before the stipendiary at Tower Bridge Police Court on December 22. The Local Taxation Committee of the County Council proceeded against Guy's Hospital "for using armorial bearings without a licence."

The prosecuting official said the Secretary had admitted that armorial bearings were used on certificates given to nurses.

For the defence it was said armorial bearings were granted to the hospital in 1725, chiefly for the purpose of being engraved on the tomb of the founder. The arms were displayed outside the hospital, and were used on the certificates granted to nurses. They were not used on notepaper, and counsel claimed that the exemption to any person who "by right of office shall wear or use the arms of any corporation" applied.

The magistrate, however, said that the Governors, who granted the certificates, were not persons who "by right of office wear or use the arms of any corporation," and he imposed a fine of 10s.

It is unfortunate that the local authorities should find it necessary to enforce, or try to, these sumptuary laws against hospitals and other charitable institutions.

